# Nanomicelle-Microsphere Composite as a Drug Carrier to Improve Lung-Targeting Specificity for Lung Cancer

**DOI:** 10.3390/pharmaceutics14030510

**Published:** 2022-02-25

**Authors:** Qianqian Zhang, Jianwei Bao, Tijie Duan, Minxing Hu, Yuting He, Junwei Wang, Rongfeng Hu, Jihui Tang

**Affiliations:** 1 School of Pharmacy, Anhui Medical University, Hefei 230032, China; zhangqianqian904@163.com (Q.Z.); baojianwei@stu.ahmu.edu.cn (J.B.); 1945010426@stu.ahmu.edu.cn (T.D.); 2045010725@stu.ahmu.edu.cn (M.H.); heyutin@stu.ahmu.edu.cn (Y.H.); 1730510056@stu.ahmu.edu.cn (J.W.); 2 Anhui Province Key Laboratory of Pharmaceutical Preparation Technology and Application, Key Laboratory of Xin’an Medicine, the Ministry of Education, Anhui Province Key Laboratory of Chinese Medicinal Formula, Plant Active Peptide Function Food Innovative Manufacturing Industry Innovation Team, Anhui University of Chinese Medicine, Hefei 230038, China

**Keywords:** micelles, microsphere, polyamine transport system, targeted drug delivery system, lung cancer

## Abstract

Lung cancer is the second-most common cancer and has the highest mortality among all cancer types. Nanoparticle (NP) drug delivery systems have been used to improve the therapeutic effectiveness of lung cancer, but rapid clearance and poor targeting limit their clinical utility. Here, we developed a nanomicelle-microsphere composite, in which doxorubicin (DOX) was loaded with spermine (Spm) modified poly (ethylene glycol)-poly(ε-caprolactone) (PEG-PCL) micelles, and then the nanomicelles were noncovalently adsorbed on the surface of poly (lactic-co-glycolic acid) (PLGA) microspheres. The attachment was confirmed by scanning electron microscopy and confocal microscopy. In vitro cell experiments, MTT assays and intracellular uptake assays were used to demonstrate the cytotoxicity and the cellular uptake of micelles in A549 cells. In vivo biodistribution studies were conducted, an orthotopic lung cancer implantation model based on C57BL/6 mice was established, and then real-time fluorescence imaging analysis was used to study the targeted efficacy of the complex. A nanomicelle-microsphere composite was successively constructed. Moreover, Spm-modified micelles significantly enhanced cytotoxicity and displayed more efficient cellular uptake. Notably, an orthotopic lung cancer implantation model based on C57BL/6 mice was also successively established, and in vivo biodistribution studies confirmed that the complex greatly improved the distribution of DOX in the lungs and displayed notable tumor targeting. These results suggested that the nanomicelle-microsphere composite has potential application prospects in the targeted treatment of lung cancer.

## 1. Introduction

Lung cancer is the second-most common malignant tumor in the world, with the highest mortality among all types of cancers [1]. It is histologically divided into SCLC (small cell lung cancer) and NSCLC (non-small cell lung cancer), of which NSCLC accounts for more than 85% of lung cancer patients worldwide. The 5-year survival rate for lung cancer patients is only 4% to 17% depending on stage and regional differences [2,3]. Unfortunately, most lung cancers are usually diagnosed at an advanced stage, with local tumor invasion or distant metastasis, which is not suitable for surgery [4]. Chemotherapy plays an important role in the current treatment of lung cancer, but its lack of selectivity leads to serious side effects. For example, doxorubicin (DOX) is one of the most effective antitumor drugs, but its clinical application is greatly restricted by serious cardiac and bone marrow toxicity [5].

Nanoparticles (NPs) have attracted extensive attention as highly promising carriers for the targeted delivery of drugs. Among them, polymer micelles, which are self-assembled NPs with core-shell structures made of amphiphilic polymers, have been widely adopted in recent years. A large number of studies have shown that polymer micelles can effectively encapsulate hydrophobic drugs into the hydrophobic nucleus to improve the water solubility of drugs [6,7], have good biocompatibility, prolong the blood circulation time, passively target and accumulate in tumor tissues via the enhanced permeability and retention (EPR) effect, actively target tumor cells after functionalized modification and represent an environmentally sensitive targeted system [8,9]. Among them, the EPR effect describes a common pathophysiological phenomenon and mechanism; namely, that the intercellular aperture of endothelial cells in tumor vessels is larger than that of normal vessels, and the lymphatic drainage of tumor cells is poor, which makes the accumulation of nanoparticles in tumor easier.

Natural polyamines, including spermidine (Spd), spermine (Spm) and putrescine (Put), are a class of biological small molecules widely found in mammalian cells which are essential for cell growth, differentiation and other physiological processes [10]. Numerous studies have shown that the majority of cancer cells have a highly expressed polyamine transport system (PTS) in the form of a transmembrane channel which inputs polyamines to maintain their rapid growth [11,12]. As a result, PTS has been considered to be a promising tumor targeting site. Indeed, several studies have examined conjugated polyamines with chemotherapeutic agents, showing that polyamine ligands significantly improved the cellular uptake efficiency mediated by PTS and enhanced cytotoxicity to cancer cells [13,14,15]. Therefore, it is worth studying polyamines as targeted ligands for active targeted systems.

However, the reported targeting effect of nano-targeted drug delivery systems is not ideal: these systems include active targeted systems or environmentally sensitive targeted systems. Only 0.7% of the administered dose is distributed in solid tumors [16,17]. One of the main reasons for this is that NPs are often obviously limited by the rapid clearance of the reticuloendothelial system (RES). The active targeted system was designed to target tumor cells or tumor tissues, but unfortunately it cannot overcome the phagocytosis of the immune system. Before they arrived at tumor cells or tumor tissue, NPs were cleared by the RES. Therefore, it is very important to prevent the rapid clearance of RES and enable NPs to reach tumor cells or tumor tissues effectively; in other words, for the first step, we should send the nano-targeted drug delivery system near the tumor tissue or tumor cells, where it can then serve to deliver targeted drugs.

Several strategies have been proposed to address this limitation. The most commonly used strategy includes the utilization of NPs modified by hydrophilic polymers such as polyethylene glycol (PEG) and poloxamer molecules [18]. However, modification with PEG accelerates blood clearance (the ABC phenomenon) and hinders uptake by targeted cells [19,20]. Using self-recognition ligands such as CD47-modified NPs to avoid the rapid clearance of RES is a novel method [21]. Another method, using the noncovalent adsorption of NPs on the surface of red blood cells (RBCs), significantly prolongs the blood circulation time and increases accumulation in the lungs [22,23,24]. However, the isolation of RBCs from whole blood is complicated, and RBCs are usually unstable in vitro. To overcome the limitations of RBCs, we have explored the idea of combining nanomicelles with microspheres: in particular, PLGA microspheres. PLGA microspheres have been extensively studied and widely used in clinical practice as drug delivery carriers, which confers the advantages of controlled and continuous release of therapeutic drugs and delivery of drugs to different target sites due to specific particle sizes and targeting ligand modifications [25,26]. In general, microparticles larger than 7 μm in diameter rapidly concentrated in the lungs through mechanical filtration of the pulmonary capillary bed after intravenous administration, thereby achieving passive lung-targeted drug delivery [25,27,28].

In the present study, the Spm-modified nanomicelles were noncovalently adsorbed on the surface of PLGA microspheres larger than 7 μm. Our aim is that more drug-loaded nanomicelles accumulate in the lungs with the delivery of microspheres and subsequently target tumor cells. In this system, the attached micelles were first accumulated efficiently to the lungs, and then their detachment from the microspheres was likely attributed to the squeezing of the complex while passing through pulmonary capillaries with diameters smaller than itself [22,24,29]. The detached micelles accumulated in tumor sites and entered tumor cells through the EPR effect and the active targeting of Spm. Overall, the nanomicelle-microsphere complex was a multistep targeted drug delivery system that increased drug accumulation in lung tumors, thereby improving anti-lung cancer efficacy and minimizing adverse reactions.

## 2. Materials and Methods

### 2.1. Materials

N-(3-Dimethylaminopropyl)-N-ethylcarbodiimide hydrochloride (EDC), N-hydroxysuccinimide (NHS), dimethyl sulfoxide (DMSO), N, N′-dimethylformamide (DMF), spermine (Spm) and doxorubicin hydrochloride (DOX·HCL) were obtained from Aladdin (Shanghai, China). HOOC-poly (ethylene glycol)_2000_-poly(ε-caprolactone)_2000_ (HOOC-PEG-PCL) was purchased from Xi’an Ruixi Biological Technology Co., Ltd. (Xi’an, China). Carboxylated poly (lactic-co-glycolic acid) (COOH-PLGA) (MW: 15,000; lactic acid: glycolic acid = 50:50) was obtained from Jinan Daigang Biomaterial Co., Ltd. (Jinan, China). Poly (ethylene-alt-maleic anhydride) (PEMA, MW: 100,000–500,000) was purchased from BIOBERRY, Inc. (Dover, DE, USA). Polyvinyl alcohol 1788 (PVA) was obtained from Aladdin (Shanghai, China). RPMI-1640 medium and Dulbecco’s modified Eagle’s medium (DMEM) were obtained from HyClone (Logan, UT, USA). Fetal bovine serum (FBS) was purchased from Biological Industries, Chemicals and Biotechnology (Kibbutz Beit Haemek, Israel). Penicillin-streptomycin (100 U/mL penicillin, 100 μg/mL streptomycin) was obtained from Beyotime Institute of Biotechnology (Shanghai, China). 3-(4,5-Dimethyl-2-thiazolyl)-2,5-diphenyl-2H-tetrazolium bromide (MTT) and 4,6-diamidino-2-phenylindole (DAPI) were obtained from Sigma (Shanghai, China).

Human lung carcinoma (A549) cells and Lewis lung carcinoma (LLC) cells were provided by Procell Life Science & Technology Co., Ltd. A549 cells were cultured in RPMI-1640, and LLC cells were cultured in DMEM. Both media were supplemented with 10% (*v*/*v*) FBS and 1% (*v*/*v*) penicillin-streptomycin. The cells were cultured in an incubator containing 5% CO_2_ at 37 °C.

C57BL/6 mice (male, 20–25 g, 4–5 weeks old) were supplied by the laboratory animal center of Anhui Medical University. The animals involved in this study were treated according to the protocols evaluated and approved by the ethical committee of Anhui Medical University (approval number: LLSC 20200715). All experiments were carried out on animals in accordance with the Guide for the Care and Use of Laboratory Animals (NIH Publications No. 8023, revised 1978).

### 2.2. Synthesis of Spm-PEG-PCL Copolymer

The polymer Spm-PEG-PCL was synthesized by carbodiimide-mediated amidation with EDC and NHS [30]. Briefly, HOOC-PEG-PCL (100 mg) was dissolved in 2 mL of DMSO, and then the carboxyl group of HOOC-PEG-PCL was activated with EDC and NHS (1:4:4 molar ratios of HOOC-PEG-PCL, EDC and NHS) for 24 h. Then, Spm (1:4 molar ratio of HOOC-PEG-PCL and Spm) was added, and the pH of the solution was adjusted to 8.0 with triethylamine. After reaction for 3 days, the resulting mixture was transferred into a dialysis bag (MWCO = 3500 Da) and dialyzed against deionized water for 48 h. Next, the product was lyophilized and stored at −20 °C.

### 2.3. Characterization of the Polymers

^1^H-NMR spectroscopy of all samples was performed by an AVANCE III 400 superconducting Fourier nuclear magnetic resonance spectrometer (Bruker, Germany) at 400 MHz using deuterated chloroform (CDCl_3_) as the solvent. FT-IR spectroscopy was executed on a Nicolet 8700 Fourier transform infrared spectrometer (Thermo Fisher Scientific, Waltham, MA, USA), and the samples were prepared by the potassium bromide tableting method.

### 2.4. Preparation of DOX-Loaded Micelles

Hydrophobic DOX was obtained by adding 20 μL of triethylamine (TEA) into DOX·HCL solution (5 mg DOX·HCL dissolved in 1 mL DMF) followed by stirring overnight in the dark. Then, Spm-PEG-PCL or HOOC-PEG-PCL (20 mg) was dissolved in the above solution. Subsequently, the mixture was added dropwise into 3 mL of PBS (0.01 M, pH 7.4) under stirring. The resulting solution was violently stirred for 1 h, treated with ice bath ultrasound for 20 min, and then dialyzed overnight against PBS (0.01 M, pH 7.4). Next, the collected product was filtered through a 0.45 μm membrane to remove unloaded DOX.

### 2.5. Characterization of Micelles

The particle size, particle size distribution and zeta potential of the micelles (in PBS, 0.01 M, pH 7.4) were measured by dynamic light scattering (DLS) using a Zetasizer Nano-ZS90 (Malvern, UK). The morphology of the micelles was observed by transmission electron microscopy (TEM, HT-7700, Tokyo, Japan). The micelle solution was dropped onto a 300-mesh copper grid with a carbon film and then stained with 2.0% (*v*/*v*) phosphotungstic acid for 30 min. The excess liquid was removed from the edge of the copper grids with filter paper. Finally, the micelle solution was air-dried at room temperature and then observed by TEM.

### 2.6. Drug Loading Capacity (LC) and Encapsulation Efficiency (EE) of Micelles

The drug loading capacity (LC) and encapsulation efficiency (EE) of the micelles were determined using UV-vis spectrophotometry at a wavelength of 482 nm. DOX-loaded micelles were destroyed and diluted with DMSO before analysis. Due to its poor solubility (the saturated solubility of free DOX in PBS (pH 7.4)was about 20 µg/mL), the free drug in the preparation was negligible. Therefore, in this study, the loaded DOX means the total drug amount in the micelles. LC (%) and EE (%) were calculated according to the following formulae:

(1) $$\mathrm{LC}\;\left(\%\right)=\frac{\mathrm{weight}\;\mathrm{of}\;\mathrm{loaded}\;\mathrm{DOX}\;\mathrm{in}\;\mathrm{the}\;\mathrm{micelles}}{\mathrm{weight}\;\mathrm{of}\;\mathrm{micelles}}\;\times \;100\%$$

(2) $$\mathrm{EE}\;\left(\%\right)=\frac{\mathrm{weight}\;\mathrm{of}\;\mathrm{loaded}\;\mathrm{DOX}\;\mathrm{in}\;\mathrm{the}\;\mathrm{micelles}}{\mathrm{total}\;\mathrm{weight}\;\mathrm{of}\;\mathrm{DOX}\;\mathrm{added}}\;\times \;100\%$$

### 2.7. Preparation of PLGA Microspheres

The well-known emulsion-solvent evaporation method was used for the preparation of PLGA microspheres [28,31]. Briefly, 100 mg of COOH-PLGA was dissolved in 1 mL of dichloromethane. Subsequently, the COOH-PLGA solution was injected into 10 mL of 1% (*w*/*v*) PEMA and 0.5% (*w*/*v*) PVA mixed solution and homogenized (Ultra Turrax T 10 basic, IKA, Staufen, Germany) at 10,000× *g* for 2 min to form the initial emulsion. The emulsion was quickly dispersed into 40 mL of ultrapure water and stirred at 40 °C for 4 h to remove the organic solvents. Next, the microspheres were washed with ultrapure water 3 times and collected by centrifugation at 3000× *g* for 3 min. The obtained microspheres were flash frozen at −140 °C and then lyophilized. Finally, the lyophilized microspheres were stored at −20 °C. The particle size and zeta potential of the micelles were measured by dynamic light scattering (DLS) using Mastersizer 3000 and Zetasizer Nano-ZS90 (Malvern Instruments, Malvern, UK).

### 2.8. Combination of the Nanomicelles and Microspheres Characterization of Micelles

One hundred milligrams of lyophilized microspheres were added to 3 mL of Spm-PEG-PCL/DOX micelle solution (PBS, 0.01 M, pH 7.4). The mixture was stirred at low speed for 6 h at room temperature [32,33]. Subsequently, the mixture was centrifuged at 3000× *g* for 1 min: the supernatant contained unadsorbed DOX micelle solution, and the precipitate was the nanomicelle-microsphere complexes. After adding a certain volume of DMSO to dissolve the complex, the amount of DOX micelles adsorbed into the microspheres was quantified by UV-vis spectrometry, and the adsorption efficiency (AE) and the drug loading capacity (LC) were calculated according to the following equation:

(3) $$\mathrm{AE}\;\left(\%\right)=\frac{\mathrm{the}\;\mathrm{amount}\;\mathrm{absorbed}\;\mathrm{DOX}}{\mathrm{the}\;\mathrm{initial}\;\mathrm{amount}\;\mathrm{of}\;\mathrm{DOX}\;\mathrm{added}}\;\times \;100\%$$

(4) $$\mathrm{LC}\;\left(\%\right)=\frac{\mathrm{the}\;\mathrm{amount}\;\mathrm{absorbed}\;\mathrm{DOX}}{\mathrm{weight}\;\mathrm{of}\;\mathrm{microspheres}}\;\times \;100\%$$

### 2.9. Characterization of the Microspheres and Nanomicelle-Microsphere Complex

The lyophilized microspheres were dispersed with an appropriate amount of ultrapure water. The mean size and size distribution of the microspheres were analyzed by a Mastersizer 3000 (Malvern Instruments, Malvern, UK). In addition, the zeta potential of the microspheres and nanomicelle-microsphere complex was analyzed by a Zetasizer Nano ZS90.

The surface morphology of the microspheres and nanomicelle-microsphere complex was detected using scanning electron microscopy (ZEISS GeminiSEM 500, Oberkochen, Germany). Briefly, lyophilized particles were attached to specimen stubs using double-sided carbon conductive tape and then sputter-coated with gold-palladium in an argon atmosphere using an SCD 050 sputter coater (BAL-TEC Instruments, Los Angeles, CA, USA) at 25 mA for 40 s. In addition, confocal laser scanning microscopy (CLSM) was also used to observe the morphology of microspheres and nanomicelle-microsphere complexes. They were dispersed in PBS and then transferred into 25-mm glass bottom dishes for observation by CLSM.

### 2.10. In Vitro Drug Release 

The drug release of micelles and nanomicelle-microsphere complexes was determined by dynamic membrane dialysis. One milliliter of the DOX-loaded micelle solution or the nanomicelle-microsphere complex suspension (500 μg DOX equivalent) was transferred into dialysis bags (MWCO = 3500 Da) and then immersed in 100 mL of PBS (pH 7.4, 0.01 M) containing 0.1% (*v*/*v*) Tween 80 [34]. The release system was incubated at 37 °C with stirring at 100 rpm under dark conditions. At predetermined time intervals (1, 2, 4, 6, 8, 10, 12, 24, 36, 48, 60 and 72 h), 2 mL of release medium was removed and replenished with an equal volume of fresh medium. Using a fluorescence spectrophotometer (Shimadzu RF-5301, Shimadzu, Japan) with an excitation wavelength of 502 nm and an emission wavelength of 557 nm, the concentration of DOX was determined against a standard calibration curve. The release studies were performed in triplicate.

### 2.11. Detachment of the Nanomicelles from PLGA Microspheres

The nanomicelle-microsphere complexes were dispersed in 1 mL PBS containing 0.1% (*v*/*v*) Tween 80 and then stirred magnetically at 1000 rpm, which was equivalent to 10 mmHg of shear stress (lung capillary wedge pressure ranging from 6 to 15 mmHg). At the set times (5, 15, and 30 min), the complex suspension was centrifuged, and the amount of DOX released from the complex was measured by fluorescence spectrophotometry. Then, the same volume of PBS was added to continue stirring. 

### 2.12. Cytotoxicity Assays 

The cytotoxicity of blank carriers, free DOX·HCL, PEG-PCL/DOX micelles and Spm-PEG-PCL/DOX micelles was assessed by MTT assay against A549 cells. In brief, A549 cells were plated in 96-well plates at a density of 1×10^4^ cells/well and then cultured for 24 h. Thereafter, drug-free Spm-PEG-PCL micelles with different carrier concentrations (1, 5, 25, 50 and 100 μg/mL) were added to the cells, and the cells were treated with DOX·HCL, PEG-PCL/DOX micelles and Spm-PEG-PCL/DOX micelles at different concentrations of DOX (0.2, 1, 5, 10 and 20 μg/mL). Untreated cells were used as the control group, and a blank group was set. After a further 24 h of incubation, the medium was discarded, and the cells were carefully washed with PBS. Next, 200 μL of fresh culture medium and 20 μL of MTT solution (5 mg/mL) were added to each well, and the cells were incubated for another 4 h. Subsequently, the culture media was replaced with 150 µL of DMSO to dissolve the formazan crystals, and the plates were gently shaken in the dark for 10 min. The absorbance of samples at 490 nm was measured by a microplate reader (Synergy HTX, Biotek, VT, USA). The cell viability (%) was calculated as follows:

(5) $$\mathrm{Cell}\;\mathrm{viability}\;\left(\%\right)=\frac{A_{\mathrm{sample}}\;-\;A_{\mathrm{blank}}}{A_{\mathrm{control}}\;-\;A_{\mathrm{blank}}}\;\times \;100\%$$

A_sample_, A_control_ and A_blank_ represented the absorbance of sample, control and blank, respectively.

### 2.13. Cellular Uptake Study

#### 2.13.1. Flow Cytometry

The cellular uptake of PEG-PCL/DOX micelles and Spm-PEG-PCL/DOX micelles was analyzed quantitatively by flow cytometry. Briefly, A549 cells were seeded at a density of 2 × 10^5^ cells/well into 6-well plates and grown for 24 h. Then, the cells were treated with 2 mL of DOX-loaded micelles (10 μg/mL) for 0.5 h, 1 h and 2 h incubation periods. Next, the cells were washed three times with cold PBS and digested with trypsin. Afterward, the cells were harvested by centrifugation at 1000× *g* for 5 min. Finally, the cells were resuspended in 1 mL of cold PBS before analysis. The mean fluorescence intensities of the cells were determined using a flow cytometer (Beckman Coulter, Brea, CA, USA).

#### 2.13.2. Confocal Laser Scanning Microscopy

The cellular uptake of PEG-PCL/DOX micelles and Spm-PEG-PCL/DOX micelles was observed using confocal laser scanning microscopy (CLSM). A549 cells were seeded on 25-mm glass bottom dishes at a density of 1 × 10^5^ cells/well and incubated for 24 h. After that, the cells were treated with 2 mL of DOX-loaded micelles (10 μg/mL) for 0.5 h, 1 h and 2 h incubation periods at 37 °C. Then, the cells were washed three times with cold PBS to remove free micelles and fixed with 4% paraformaldehyde at 37 °C for 20 min. Next, the nuclei were stained with DAPI at 37 °C for 15 min. Finally, the cells were washed three times with cold PBS and then observed by CLSM (Zeiss LSM880, Oberkochen, Germany).

### 2.14. Mechanism of Cellular Internalization

To investigate the internalization mechanism of micelles, A549 cells were seeded on six-well plates at a density of 2 × 10^5^ cells/well. After incubating for 24 h, one group of cells was pretreated with 2 mL of Spm (10 μg/mL) for 1 h. Then the cells were washed three times with cold PBS and incubated with 2 mL of PEG-PCL/DOX micelles or Spm-PEG-PCL/DOX micelles (at an equal DOX dose of 10 μg/mL) for another 1 h at 37 °C. In addition, the other cell group was incubated at low temperature (4 °C) for 1 h, and they were then exposed to the micelles and cultured at 4 °C for another 1 h. Moreover, the cells cultured at 37 °C without pretreatment were used as the control group. After micellar treatment, the cells were washed three times with cold PBS and the mean fluorescence intensity of the cells was detected by flow cytometry.

### 2.15. Orthotopic Lung Cancer Model in Mice

LLC cells (growing in log phase) were suspended in PBS (final cell density, 1 × 10^6^ cells/mL) containing an equal volume of BD Matrigel^®^ (Corning, Corning, NY, USA) to prevent the suspension from leaking out of the lung. The cell suspension was placed on ice before injection. After C57BL/6 mice were anesthetized by intraperitoneal injection of chloral hydrate, the left chest was swabbed with 75% alcohol. Subsequently, a small skin incision (approximately 1 cm in length) was made in the left lateral dorsal axillary line, approximately 1.5 cm above the lower rib line just below the inferior border of the scapula. The skin and subcutaneous tissue were separated until the chest wall was exposed. While observing the movement of the left lung through the pleura, an insulin syringe containing LLC cells was directly inserted through the intercostal space into the lung (to a depth of 3 mm). After injection, the skin incision was closed with a surgical suture [35,36,37].

### 2.16. In Vivo Biodistribution

The tumor-bearing C57BL/6 mice inoculated with LLC cells for 14 days were randomly assigned and injected with DOX·HCL, Spm-PEG-PCL/DOX micelles and nanomicelle-microsphere complexes via the tail vein at a DOX dose of 4 mg/kg (*n* = 3). At 12 h and 24 h post injection, these mice were sacrificed, and major organs (including the heart, liver, spleen, lung and kidney) and tumors were collected for fluorescence imaging using an Ami HT in vivo imaging system (Spectral Instruments Imaging, LLC, Tucson, AZ, USA) with an excitation wavelength of 500 nm and an emission wavelength of 630 nm.

### 2.17. Statistical Analysis

All experiments were performed at least three times, and the data are expressed as the mean ± standard deviation (SD). Statistical analysis between two groups was carried out using the student’s *t*-test in SPSS 20, and *p* < 0.05 was considered statistically significant.

## 3. Results and Discussion

### 3.1. Synthesis of Spm-PEG-PCL

**Figure 1:**
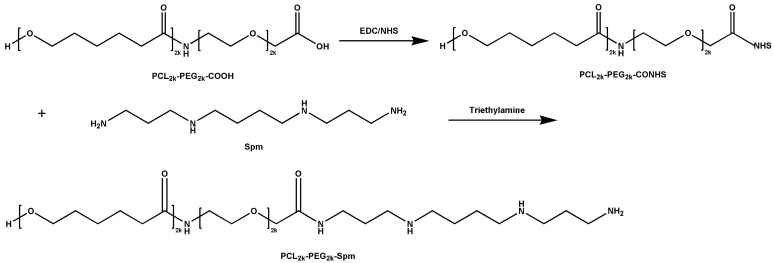
Synthesis route to Spm-PEG-PCL copolymer.

**Figure 2:**
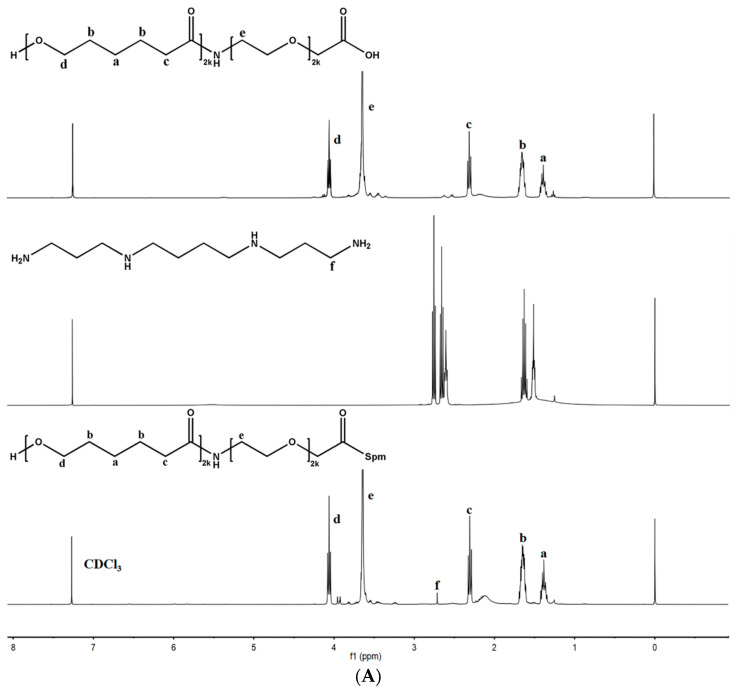

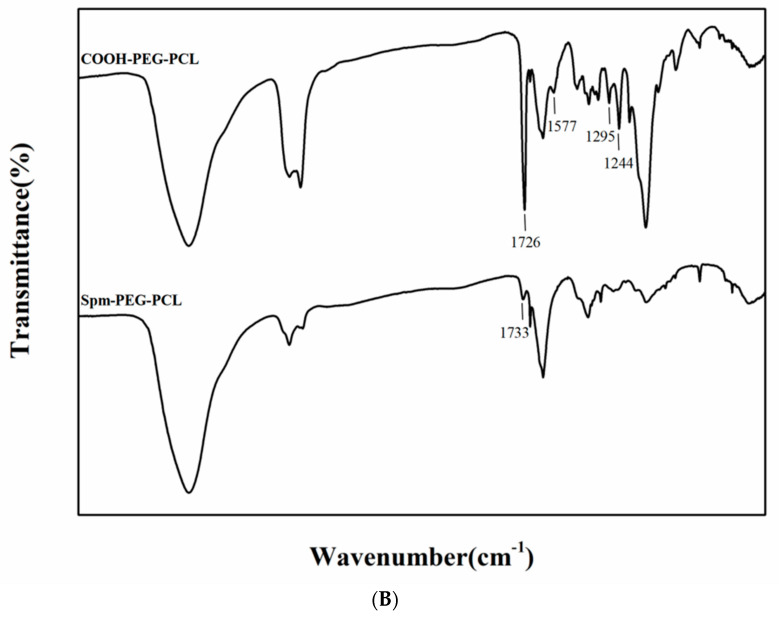
Structural confirmation with (**A**) ^1^H-NMR spectra of HOOC-PEG-PCL, Spm and Spm-PEG-PCL; (**B**) FT-IR spectra of HOOC-PEG-PCL and Spm-PEG-PCL.

The synthesis route for the conjugation of Spm-PEG-PCL is shown in Figure 1. The structure of Spm-PEG-PCL was confirmed by ^1^H-NMR spectrometry (see Figure 2A). The characteristic peaks of a (δ = 1.3 ppm), b (δ = 1.5 ppm), c (δ = 2.27 ppm), d (δ = 3.98 ppm) and e (δ = 3.51 ppm) can be assigned to the protons of the methylene groups of PCL and PEG, respectively. Finally, the modification of Spm on COOH-PEG-PCL was evidenced by the emergence of the peaks of f (δ = 2.8–2.5 ppm) that originated from the methylene protons in the Spm skeleton.

FT-IR spectroscopy was also used to characterize the synthesized polymers (see Figure 2B). COOH-PEG-PCL showed signals at 1726 cm^−1^, which belonged to the stretching vibration peaks of the carbonyl group (C=O), while Spm-PEG-PCL displayed a new absorption signal at 1733 cm^−1^. After modification with Spm, the signals at 1244 cm^−1^ and 1295 cm^−1^ (C–O stretching vibration) and the signal at 1577 cm^−1^ (–COO^−^ asymmetric stretching vibration) disappeared, which was due to the conjugation of Spm on the terminal carboxyl group of COOH-PEG-PCL.

The above ^1^H-NMR and FT-IR results indicate that Spm-PEG-PCL is successfully synthesized.

### 3.2. Characterization of the Micelles

**Figure 3:**
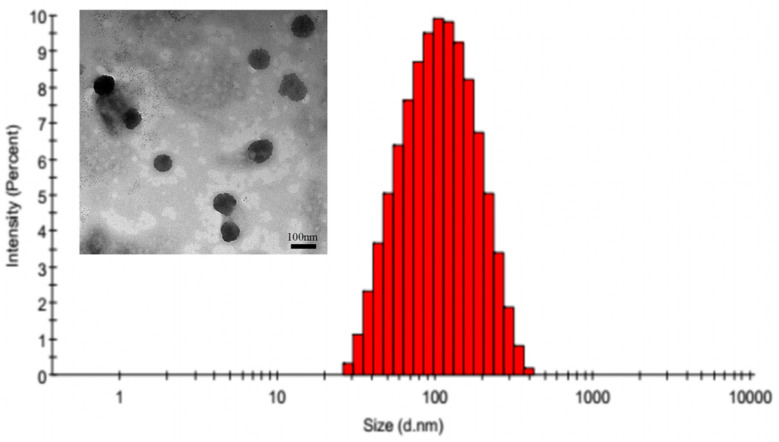
Typical size distribution determined by DLS and TEM micrograph of the Spm-PEG-PCL/DOX micelles; scale bar is 100 nm.

DOX-loaded micelles were prepared by a dialysis method. As shown in Table 1, the mean diameters of PEG-PCL/DOX and Spm-PEG-PCL/DOX were (122.93 ± 12.80) nm and (110.91 ± 9.68) nm, respectively, which were favorable for micelle aggregation in tumor tissues through the EPR effect. The surface modification of Spm had little effect on particle size but changed the zeta potential from negative (−9.26 ± 2.21) mV to positive (+6.25 ± 0.54) mV. This change also indicated the successful synthesis of Spm-PEG-PCL and can be attributed to the conjugation of the anionic carboxyl terminus of PEG with spermine, which carries a highly positively charged amino unit. In addition, the LC (%) and EE (%) of PEG-PCL/DOX micelles and Spm-PEG-PCL/DOX micelles were (13.5 ± 0.2)% and (66.3 ± 1.5)%; (13.9 ± 0.6)% and (69.3 ± 3.4)%, respectively. The TEM image in Figure 3 showed that Spm-PEG-PCL/DOX micelles did not aggregate and were morphologically homogeneous. The particle diameter observed by TEM is smaller than the particle size measured by dynamic laser scattering. This may be because the sample preparation process is different between the two methods, with the TEM photos describing the size of the sample in the dry state, while the dynamic laser scattering assay is the size of the nanoparticles in the hydration state. That is, the TEM observes the actual state of nanoparticles, and the dynamic laser scattering measurement is a diameter of hydraulic force. Therefore, in hydration, nanoparticles have a higher hydraulic volume due to swelling, so the size of the particle determined by dynamic laser scattering is greater than the TEM assay.

Polyamines have been used as targeted ligands in many drug delivery systems. For example, polyamines were used to functionalize PEGylated poly(lactide-co-glycolide) nanoparticles [15], β-cyclodextrin [38] or hydrophobic stearyl chains for tumor-targeted drug delivery [13]. Because of their positive electronic properties, polyamines usually affected the charge of the modified carrier. In DOX-loaded PEG-PLGA nanoparticles, the charge increased from −5.2 mV to −2.4 mV after spermidine modification [15], while when a hydrophobic stearyl chain is modified with spermine [13], a positive charge result.

In our study, after modification, the micelles charge changed from negative to positive, which facilitated the adsorption of nano-micelles to negatively charged microspheres by charge action. In addition to electrostatic interactions, nanoparticles also adsorbed onto microspheres via hydrophobic forces such as nanoparticles attachment to red blood cells, as reported by Anselmo et al. [29].

### 3.3. Characterization of the PLGA Microspheres and the Nanomicelle-Microsphere Complex

The particle size of microspheres is a key factor that influences their lung-targeting performance. Previous reports have demonstrated that microspheres of 7~30 μm could be mechanically intercepted by the capillary bed after intravenous injection and then accumulate in the lung [27,28]. In this study, PLGA microspheres were prepared by the (O/W) emulsification-solvent evaporation method. The average particle size of the microspheres was (11.73 ± 1.31) μm, and 90% of the microspheres were distributed in the range of 7~30 μm, which met the requirements of passive lung targeting.

With carboxylic acid groups only at the ends of polymer chains, the negative charge on the surface of PLGA-COOH microspheres is limited. Therefore, in the preparation process of PLGA microspheres, PEMA was used to increase the number of carboxyl groups on the surface of the microspheres and make the microspheres carry more negative charge [39,40,41]. PVA is used as a steric stabilizer to effectively prevent the aggregation of microspheres [42]. The zeta potential of the microspheres was (−44.33 ± 1.01) mV, indicating that the microspheres carry a large amount of negative charge. Notably, the zeta potential of the nanomicelle-microsphere complexes was transformed to (−9.99 ± 0.01) mV, suggesting the occurrence of electrostatic interactions during the adsorption process.

It has been shown that neutrally or negatively charged NPs remain stable in vivo and reduce interactions with plasma proteins and toxicity to normal tissues. However, for tumor tissue, positively charged nanoparticles are more prone to uptake by tumor cells and improve the antitumor effect. For example, Liu et al. developed a charge reversal drug delivery system, which realized the reversal of negative charge to positive charge under acidic tumor conditions. Thus, it circulates steadily in the physical environment and enhances cellular endocytosis in the acidic tumor environment [43]. With our system, the negative charge of the nanomicelle-microsphere complex will ensure its stability in vivo and reduce interaction with plasma proteins, and the positive charge of Spm-PEG-PCL micelles will make it easier to take up by tumor cells.

**Figure 4:**
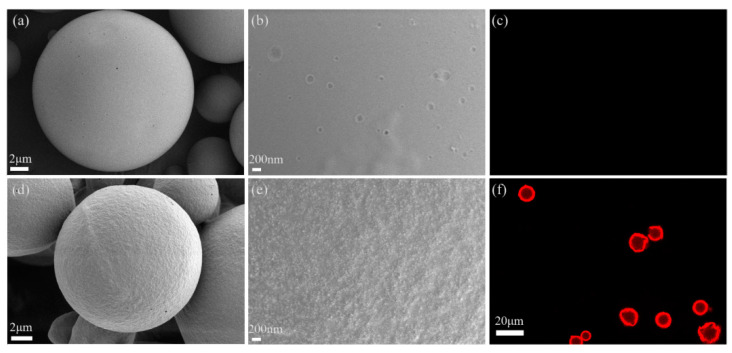
The morphologies of PLGA microspheres and the nanomicelle-microsphere complexes were observed by SEM and CLSM. (**a**) Morphology of PLGA microspheres by SEM (×4 k); scale bar is 2 μm; (**b**) Surface image of PLGA microspheres by SEM (×20 k); scale bar is 200 nm. (**c**) Morphology of PLGA microspheres by CLSM (×40 k); (**d**) Morphology of the nanomicelle-microsphere complexes by SEM (×5 k); scale bar is 2 μm; (**e**) Surface image of the nanomicelle-microsphere complexes by SEM (×20 k); scale bar is 200 nm; (**f**) Morphology of the nanomicelle-microsphere complexes by CLSM (×40 k); scale bar is 20 μm.

After the adsorption experiment, the unadsorbed micelles and adsorbed micelles–microsphere complexes were separated by centrifugal method. The morphologies of PLGA microspheres and nanomicelle-microsphere complexes were observed by SEM and CLSM. As shown in Figure 4a, the microspheres were completely spherical in shape with an average particle size of approximately 10 μm, which was basically consistent with the results measured by a Mastersizer 3000. Moreover, the surface of the microspheres was smooth (see Figure 4b), while the surface of the nanomicelle-microsphere complexes did not become smooth, mainly due to a large number of nanomicelles forming films on the surface of the microspheres (see Figure 4d,e). In the process of freeze-drying, nanomicelles are dehydrated and form a film on the surface of microspheres; thus, the particle morphology of nanomicelles cannot be seen by SEM. To further verify the adsorption of nanomicelles on the surface of microspheres, CLSM was used to observe the morphology of the complex. As shown in Figure 4c, independent PLGA microspheres showed no fluorescence; but there was strong red fluorescence around the microspheres in the nanomicelle-microsphere complexes (see Figure 4f), indicating that a large number of DOX micelles were adsorbed on the surface of PLGA microspheres. A quantitative study showed that the adsorption efficiency (AE) and the drug loading capacity (LC) of the nanomicelle-microsphere complexes were 71.85% ± 3.43% and 1.48% ± 0.19%, respectively.

### 3.4. In Vitro Drug Release

**Figure 5:**
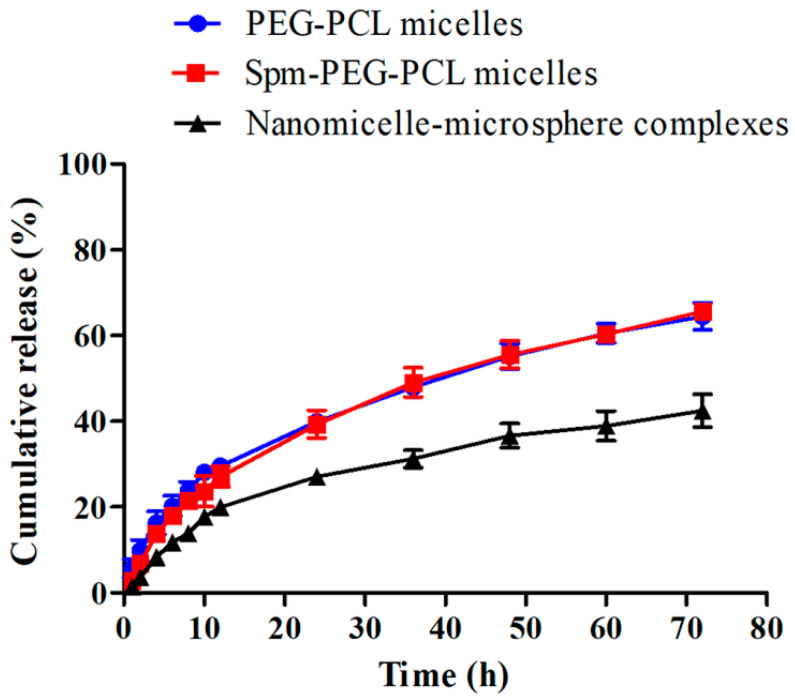
In vitro release of DOX from two micelles and the nanomicelle-microsphere complexes at pH 7.4 (*n* = 3).

The sustained-release property of drugs is also an important characteristic of drug delivery systems. In this study, the release of DOX from the micelles and the nanomicelle-microsphere complex were carried out in PBS at pH 7.4, simulating the in vivo biological environment, and the release profiles are shown in Figure 5. The total releases of DOX from PEG-PCL micelles, Spm-PEG-PCL micelles and the nanomicelle-microsphere complexes within 72 h were (64.49 ± 5.46)%, (65.58 ± 3.16)% and (42.69 ± 6.64)%, respectively, exhibiting obvious sustained drug release under physiological conditions. The drug release rate was similar between the two micelles, but it was noteworthy that the complexes released drugs more slowly than the micelles. It may be due to the micelle-DOX-microsphere interactions generated during the adsorption process, which appeared to create a diffusion barrier and induced changes in the release rate.

### 3.5. Detachment of the Micelles from PLGA Microspheres

To simulate the detachment of Spm-functionalized micelles from the surface of microspheres as the complex passes through pulmonary capillaries with diameters smaller than the complex in vivo, we conducted in vitro separation experiments and determined the desorption amount of the micelles. As shown in Table 2, the cumulative release of the nanomicelle-microsphere complex under shear was 13.9% within 30 min, which was approximately 14 times that within 1 h without shear release in vitro (see Figure 5).

The results suggested that the electrostatically and hydrophobically adsorbed targeted micelles detached rapidly from the microspheres during their passage through the tiny pulmonary capillary, which contributed to drug accumulation in lung tumors. This is similar to the noncovalent attachment of nanoparticles to red blood cells, in which the particles detached from RBCs upon exposure to physiological shear stresses experienced when RBCs pass through lung capillaries [29]. However, compared to RBCs, poly (lactic-co-glycolic acid) (PLGA) microspheres have been widely used in clinical practice, and they are considered safe, cost-effective, and easy to work with.

In this study, because the micelles are positively charged, the micelles are easily accessible to the tumor cells. In addition, the actively targeted ligands made Spm-PEG-PCL micelles more prone to uptake by tumor cells [13,15,38].

### 3.6. Cytotoxicity Assays

**Figure 6:**
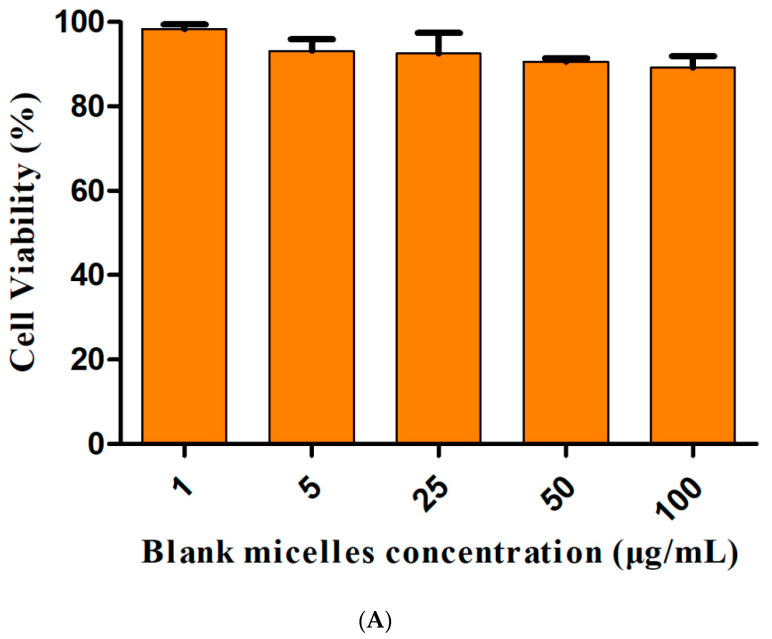

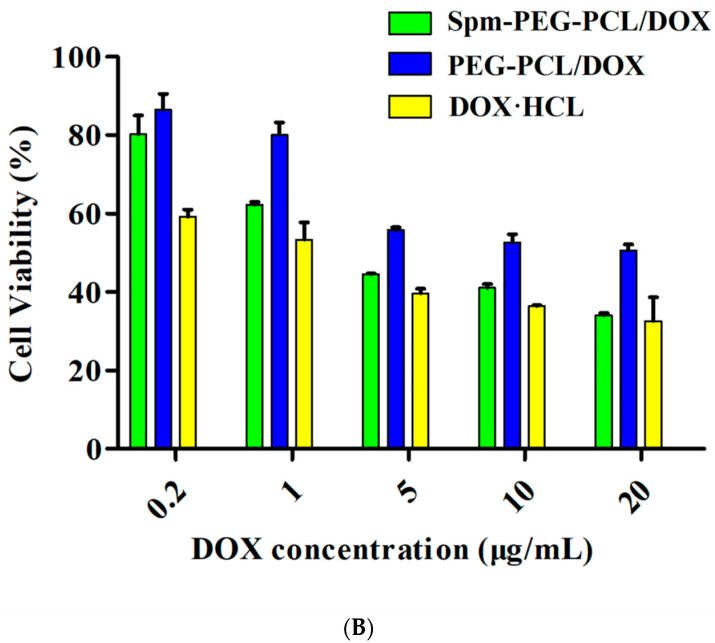
Cytotoxicity of blank and drug-loaded micelles in A549 cells (*n* = 3). (**A**) Cytotoxicity of blank Spm-PEG-PCL micelles at varying polymer concentrations. (**B**) Cytotoxicity of Spm-PEG-PCL/DOX, PEG-PCL/DOX micelles and DOX·HCL.

The in vitro cell viability of drug-free Spm-PEG-PCL micelles is shown in Figure 6A. More than 90% of the cells were viable in the concentration range of 1–100 μg/mL, showing that the carrier had good biocompatibility with A549 cells. As shown in Figure 6B and Table 3, Spm-PEG-PCL/DOX micelles exhibited higher cytotoxicity, with a half-maximal inhibitory concentration (IC_50_) value of 3.84 ± 0.51 μg/mL, which was approximately 3.9 times lower than that of PEG-PCL/DOX micelles. These results suggested that Spm-modified micelles significantly enhanced antitumor activity compared to unmodified micelles. The IC_50_ value of DOX·HCL was 1.18 ± 0.43 μg/mL, indicating that DOX·HCL possessed the highest cytotoxicity, which was mainly due to the free diffusion of DOX and the sustained release of micelles. However, when administered intravenously, free DOX is distributed throughout the body and fail to enter the tumor cells effectively. In summary, Spm-PEG-PCL/DOX micelles are predicted to work effectively, since they accumulate efficiently in tumor tissue and enter tumor cells.

### 3.7. Cellular Uptake Study

#### 3.7.1. Flow Cytometry

**Figure 7:**
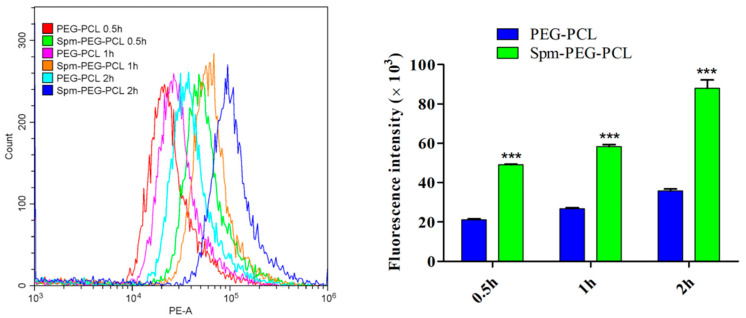
The cellular uptake of PEG-PCL/DOX and Spm-PEG-PCL/DOX micelles in A549 cells analyzed by flow cytometry (*n* = 3), *** *p* < 0.001 compared to PEG-PCL micelles.

Drug-loaded nanoparticles accumulate in the tumor site and subsequent effective cellular uptake is crucial to the drug therapeutic effect. Therefore, the cellular uptake study here was to visualize the impact of Spm ligand modification on the cellular uptake of micelles. The A549 cells were incubated with Spm-PEG-PCL micelles and PEG-PCL micelles for 0.5 h, 1 h and 2 h, respectively. As shown in Figure 7 and Table 4, the mean fluorescence intensity values of PEG-PCL micelles were 21,067.30, 26,685.73 and 35,738.67, while those of Spm-modified micelles were 49,120.83, 58,360.47 and 88,030.03, respectively.

The results indicated that the cellular uptake of the two micelles was time-dependent, and that the Spm-targeting ligand significantly improved the cellular uptake of micelles. In addition, in the nanomicelles-microsphere complex system, the microspheres simply serve as a transport vehicle to deliver the drug-loaded micelles to the lungs, so the complexes group was not designed for cytotoxicity and cellular uptake experiments.

#### 3.7.2. Confocal Laser Scanning Microscopy

**Figure 8:**
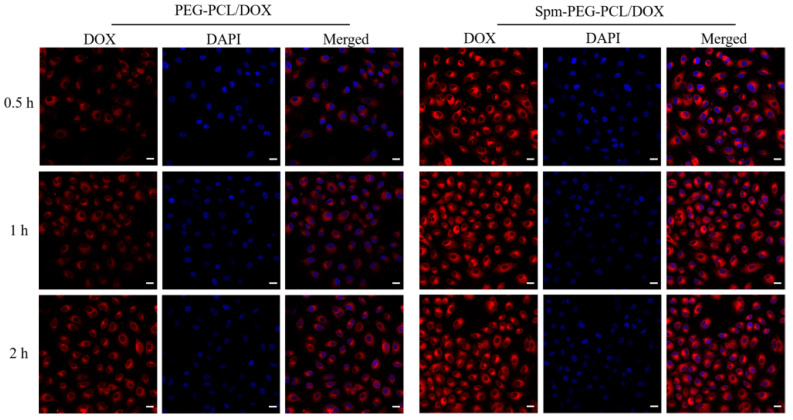
Cellular uptake of Spm-PEG-PCL/DOX and PEG-PCL/DOX micelles observed by CLSM. DOX (red) was used to label micelles, DAPI (blue) was used to stain nuclei, and the scale bar is 20 μm.

As shown in Figure 8, red represents DOX micelles, and blue represents the cell nucleus. After incubation for 0.5 h, Spm-PEG-PCL/DOX micelles showed obvious red fluorescence, indicating rapid internalization. In addition, the red fluorescence gradually intensified with longer incubation time, suggesting that the cellular uptake of micelles was time-dependent. Notably, at each timepoint, the fluorescence of Spm-PEG-PCL/DOX micelles was considerably brighter than that of PEG-PCL/DOX micelles, which confirmed that the Spm ligand increased the selective uptake of micelles.

### 3.8. Mechanism of Cellular Internalization

**Figure 9:**
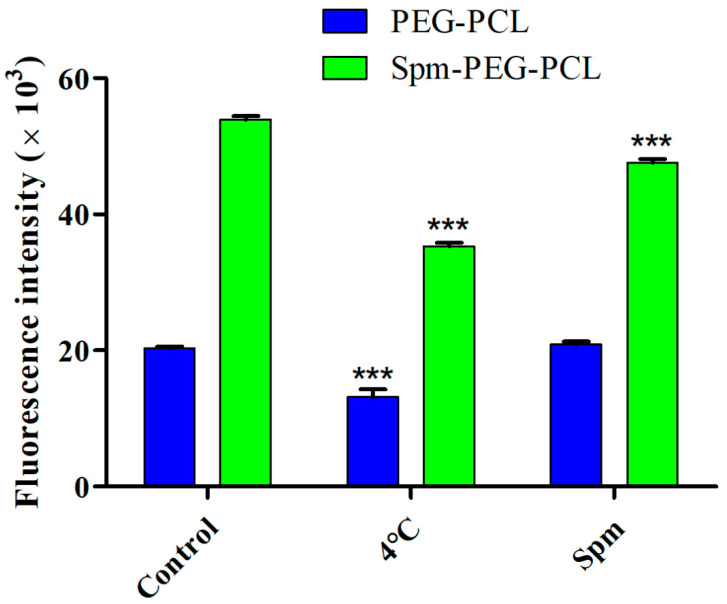
The cellular uptake mechanisms of Spm-modified micelles were studied by flow cytometry. Cellular uptake under 4 °C pretreatment or in the presence of the competitive inhibitor Spm at 37 °C for 1 h. Cells cultured without pretreatment were used as the control group (*n* = 3). *** *p* < 0.001 compared to the control group.

As shown in Figure 9 and Table 5, the cellular uptake of micelles at 37 °C was approximately 1.5-fold higher than that at 4 °C, indicating that the endocytosis pathway of micelles in A549 cells was mainly energy-dependent [44].

To confirm that the cellular uptake of Spm-modified micelles was mainly mediated by PTS, free Spm was employed to block PTS [15]. In this case, the specific enhancement effect of Spm-PEG-PCL or PEG-PCL micelles can be abolished if PTS is important for particle uptake. As shown in Table 5 and Figure 9, when pretreated with Spm, the mean fluorescence intensity of PEG-PCL micelles remained at approximately 20,000, while that of Spm-PEG-PCL micelles significantly decreased from 52,038.40 to 47,577.60. The results showed that the cellular uptake of Spm-PEG-PCL micelles pretreated with Spm was significantly lower than that of untreated controls, while the cellular uptake of PEG-PCL micelles pretreated with Spm was similar to that of untreated controls. Therefore, these results confirmed that Spm-modified micelles mainly compete with free Spm to enter tumor cells via PTS.Moreover, the uptake of Spm-PEG-PCL micelles pretreated with Spm was significantly reduced, but not to the level of PEG-PCL micelles. We think it is likely that the concentration of Spm and the pretreatment time were insufficient, so that the polyamine transporter is not completely occupied by Spm. However, since the high concentration of spermine had some cytotoxicity, we did not pretreatment cells with a high concentration of Spm.

### 3.9. In Vivo Biodistribution

**Figure 10:**
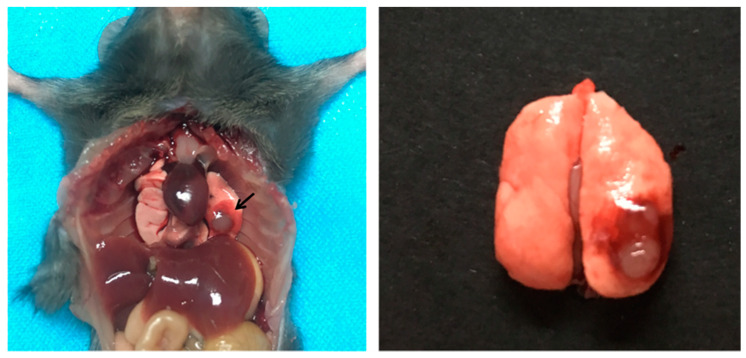
Orthotopic lung cancer model in C57BL/6 mice.

In this study, an LLC orthotopic lung cancer model was established by thoracic injection. First, LLC cells were suspended in PBS containing BD Matrigel^®^ to prevent the suspension from leaking out of the lung during inoculation and to facilitate the formation of a solitary tumor nodule in the lung parenchyma. As shown in Figure 10, 14 days after LLC cells were inoculated in C57BL/6 mice, a distinct tumor nodule developed in the left lung, which gradually grew larger over time.

Here, we used an orthotopic xenograft model rather than a subcutaneous tumor model, since the metastatic pattern of in situ tumors is similar to that of the primary tumor and can effectively predict clinical drug effects [45]. In addition, our lung targeted delivery system was designed to first target lung tissue and then to target lung tumor cells, so the in situ tumor model is more appropriate.

**Figure 11:**
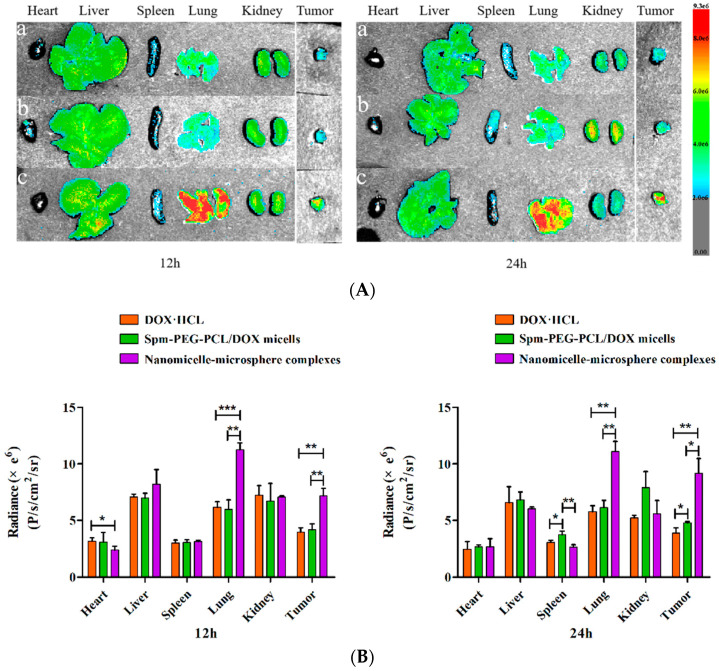
(**A**) Ex vivo fluorescent images of DOX in major organs and tumors at 12 h and 24 h after intravenous injection of (**a**) DOX·HCL, (**b**) Spm-PEG-PCL/DOX micelles and (**c**) nanomicelle-microsphere complexes at a DOX dosage of 4 mg/kg; (**B**) Radiance of the three groups of lungs and tumors (*n* = 3). * *p* < 0.05, ** *p* < 0.01 and *** *p* < 0.001.

To visualize the tissue distribution and tumor accumulation in LLC tumor-bearing C57BL/6 mice, the mice were injected with free DOX·HCL, DOX-loaded Spm-PEG-PCL micelles and the nanomicelle-microsphere complex through the tail vein, and then imaging was performed using a fluorescence imaging system at 12 h and 24 h. Figure 11A shows ex vivo DOX fluorescence images of tumors and major organs (including the heart, liver, spleen, lung, and kidney) isolated from C57BL/6 mice at 12 h and 24 h after injection, and the ex vivo mean fluorescence intensity was shown in Figure 11B. As shown in Figure 11B, the tumor mean fluorescence intensity of the Spm-PEG-PCL micelle group was 1.24-fold higher than that of the DOX·HCL group at 24 h, indicating that Spm-modified micelles have effective tumor-targeting efficiency. This phenomenon was mainly due to the EPR effects afforded by the PEGylated micelle surface and the binding affinity of the Spm-modified micelles to tumor cell surface receptors [15]. Notably, as shown in Figure 11A, compared with DOX·HCL and the micelles, the nanomicelle-microsphere complex group showed much stronger DOX fluorescence on lungs and tumors than on the other organs at 12 h and 24 h. Figure 11B showed that the lung mean fluorescence intensity of the complex group was, respectively, 1.88- and 1.82-fold higher than that of the micelles and the DOX·HCL group, and the tumor mean fluorescence intensity of the complex group was, respectively, 1.72- and 1.80-fold higher than of the micelles and the DOX·HCL group at 12 h. At 24 h, the lung mean fluorescence intensity of the complex group was 1.81 and 1.92 times higher than that of the micelles and DOX·HCL groups, and the tumor mean fluorescence intensity of the complex group was 1.92 and 2.37 times higher than that of the micelles and DOX·HCL groups, respectively. These results demonstrated that the micelles attached to the surface of PLGA microspheres were effectively delivered to and accumulated within the lungs and tumors, thus improving lung targeting and tumor selectivity. This is similar to that reported in the literature [29]. In addition, the heart mean fluorescence intensity of the complex group was 1.32-fold lower than that of DOX·HCL groups at 12 h, which would help to reduce the cardiac toxicity of DOX. In the system, NPs were adsorbed on the surface of PLGA microspheres, and then delivered to the lungs via the microspheres. Subsequently, the NPs detached from the microspheres surface as they passed through the pulmonary capillaries and were mechanically constricted by the lungs. Finally, the detached NPs were taken up effectively by lung tumor cells due to the EPR effects and PTS-directed active targeting effects.

## 4. Conclusions

In summary, Spm-functionalized nanomicelles were noncovalently and reversibly absorbed on the surface of PLGA microspheres, constructing a multistep lung-targeted drug delivery system. Upon intravenous administration, the complexes were trapped by pulmonary capillaries and accumulated in the lungs, and then the attached micelles desorbed, likely due to the squeezing of the complex when passing through tiny pulmonary capillaries. In vitro cell studies, Spm-modified micelles significantly enhanced cytotoxicity and promoted the cellular uptake through specific binding to PTS. Furthermore, in vivo experiments in LLC orthotopic lung cancer-bearing mice confirmed that the micelles which were attached to the surface of PLGA microspheres greatly improved the drug accumulation in the lungs and tumors. Taken together, the nanomicelle-microsphere complex improved the targeting efficiency of DOX through the combination of passive and active targeting mechanisms, providing great potential for targeted lung cancer therapy.

## Figures and Tables

**Table 1:** Characterization of PEG-PCL/DOX and Spm-PEG-PCL/DOX micelles (mean ± standard deviation, *n* = 3).

Sample	Size (nm)	PDI	Zeta Potential (mV)	LC (%)	EE (%)
PEG-PCL/DOX	122.93 ± 12.79	0.20 ± 0.01	−9.26 ± 2.21	13.46 ± 0.20	66.27 ± 1.50
Spm-PEG-PCL/DOX	110.91 ± 9.68	0.19 ± 0.01	+6.25 ± 0.54	13.91 ± 0.64	69.34 ± 3.37

**Table 2:** Drug release stimulated through pulmonary capillary constriction (mean ± standard deviation, *n* = 3).

T (min)	DOX Release (%)	Accumulated DOX Release (%)
5	7.95 ± 1.15	7.95 ± 1.15
15	3.86 ± 0.52	11.81 ± 1.60
30	2.10 ± 0.76	13.90 ± 1.90

**Table 3:** IC_50_ values of different preparations (mean ± standard deviation, *n* = 3).

Treatment Compositions	PEG-PCL/DOX	Spm-PEG-PCL/DOX	DOX·HCL
IC_50_	14.86 ± 2.54	3.84 ± 0.51 **	1.18 ± 0.43

** *p* < 0.01 compared to PEG-PCL/DOX micelles. IC_50_, half-maximal inhibitory concentration.

**Table 4:** Flow cytometry data for the efficiency of micelle uptake (mean ± standard deviation, *n* = 3).

Sample	0.5 h	1 h	2 h
PEG-PCL	21,067.30 ± 434.41	26,685.73 ± 648.97	35,738.67 ± 999.51
Spm-PEG-PCL	49,120.83 ± 287.79 ***	58,360.47 ± 1046.97 ***	88,030.03 ± 4192.13 ***

*** *p* < 0.001 compared to PEG-PCL micelles.

**Table 5:** Flow cytometry data for the mechanisms of micelle uptake (mean ± standard deviation, *n* = 3).

Sample	Control	4 °C	Spm
PEG-PCL	20,327.90 ± 230.95	13,754.80 ± 335.41 ***	20,890.97 ± 394.37
Spm-PEG-PCL	52,038.40 ± 698.02	35,277.60 ± 534.46 ***	47,577.60 ± 527.92 ***

*** *p* < 0.001 compared to control.

## Data Availability

Not applicable.

## Abbreviations

Abbreviations	Full names
CLSM	Confocal laser microscopy
DLS	Dynamic light scattering
DMF	Dimethylformamide
DMSO	Dimethyl sulfoxide
DOX·HCL	Doxorubicin Hydrochloride
EE	The drug encapsulation efficiency
EPR effect	The enhanced permeability and retention effect
FBS	Fetal Bovine Serum
FCM	Flow cytometry
IC50	Half maximal inhibitory concentration
LC	The drug loading capacity
LLC cells	Lewis lung carcinoma cells
NPs	Nanoparticles
PBS	Phosphate buffer saline
PEG-PCL	Poly (ethylene glycol)-Poly (ε-caprolactone)
PEMA	Poly(ethylene-alt-maleic anhydride)
PLGA	Poly (lactic-co-glycolic acid)
PTS	Polyamine transport system
PVA	Poly(vinyl alcohol)
RSD	Relative standard deviation
SEM	Scanning electron microscopy
Spm	Spermine
TEM	Transmission electron microscopy
